# Design of Acoustic Bifocal Lenses Using a Fourier-Based Algorithm

**DOI:** 10.3390/s21248285

**Published:** 2021-12-11

**Authors:** José Miguel Fuster, Sergio Pérez-López, Pilar Candelas

**Affiliations:** Centro de Tecnologías Físicas, Universitat Politècnica de València, 46022 València, Spain; serpelo1@teleco.upv.es (S.P.-L.); pcandelas@fis.upv.es (P.C.)

**Keywords:** zone plate, binary sequence, Fourier transform, signal processing, acoustic focusing

## Abstract

In this work, we develop a new design method based on fast Fourier transform (FFT) for implementing zone plates (ZPs) with bifocal focusing profiles. We show that the FFT of the governing binary sequence provides a discrete sequence of the same length, which indicates the location of the main foci at the ZP focusing profile. Then, using reverse engineering and establishing a target focusing profile, we are capable of generating a binary sequence that provides a ZP with the desired focusing profile. We show that this design method, based on the inverse fast Fourier transform (IFFT), is very flexible and powerful and allows to tailor the design of bifocal ZPs to achieve focusing profiles with the desired foci locations and resolutions. The key advantage of our design algorithm, compared to other alternatives presented in previous works, is that our method provides bifocal focusing profiles with an absolute control of the foci locations. Moreover, although we analyze the performance of this novel design algorithm for underwater ultrasonics, it can also be successfully extended to different fields of physics, such as optics or microwaves, where ZPs are widely employed.

## 1. Introduction

Acoustic focusing is widely employed to explore objects or biological tissues where optical techniques are not optimum due to scattering and absorption phenomena. Ultrasound focusing applications include non-destructive testing in industrial scenarios [1] and biomedical imaging in medical applications [2]. In the past years, several methods have been introduced to focus ultrasound waves, such as holographic lenses or acoustic metasurfaces. Holographic lenses allow to focus the acoustic intensity into complex arbitrary 3D shapes by means of a simple 3D printed phase-plate [3,4,5,6]. Alternatively, acoustic metasurfaces or metamaterials can focus acoustic energy by shaping the incident wavefront into targeted complex amplitude and phase pressure distributions. The unit cells of the metamaterial can be designed with different techniques, such as Helmholtz resonators [7], subwavelength slits [8], or coiling-up space channels [9,10,11]. Despite the high versatility of both holographic lenses and acoustic metasurfaces, these kind of structures usually require a complex design process with optimization algorithms and 3D wave propagation numerical simulations to achieve the desired pressure pattern.

In contrast, Fresnel Zone Plates (FZPs) are planar monofocal lenses that can achieve good focusing performance while keeping an easy design and manufacturing process. This type of lenses has been widely employed in the past in various fields, including microwaves [12], optics [13,14], and acoustics [15,16,17]. Fresnel ZPs are built by concatenating a series of concentric rings of decreasing width. Each ring is known as a Fresnel region, and its width and inner and outer radii depend on various parameters, such as the focal distance and the operating frequency of the lens. The different Fresnel regions can be implemented by alternating either pressure blocking (opaque) with transparent regions, which is known as a Soret zone plate (SZP), or by alternating phase-reversal with transparent regions, which results in a Phase-Reversal zone plate (PRZP).

Interestingly, in recent years, a novel kind of lenses based on applying a binary sequence to the different Fresnel regions of a conventional Fresnel ZP [18] have been introduced. Depending on the nature of this binary mask, different focusing properties are obtained. For instance, using a M-bonacci binary mask results in a bifocal profile with two equal-intensity foci [19,20,21], while using fractal Cantor sequences provides a focusing profile with interesting self-similarity multifocal properties [22,23,24,25]. Thue-Morse binary sequences combine both effects and provide focusing profiles with bifocal fractal properties [26,27].

Thus, the use of binary sequences is very appealing in the design of ZPs, because they provide a high versatility in the design of the focusing profile. Although M-bonacci binary sequences have been successfully used to provide bifocal focusing profiles, they present some limitations in the control of the foci location, as both foci are related by a fixed parameter determined by the order of the M-bonacci sequence. For example, in the case of Fibonacci sequences, the ratio between both foci is $z_{2}/z_{1}=1/\left(\varphi_{2}-1\right)$, with $\varphi_{2}=\left(1+\sqrt{5}\right)/2$ being the golden mean. Other alternative methods for providing bifocal profiles based on combining preexisting Fresnel ZPs have also been proposed in [28], but they require a complex design process with an accurate control of the phase interference pattern.

In this work, we propose a simple design technique based on the fast Fourier transform (FFT) capable of providing ZPs with two equal-intensity foci located at arbitrary distances. The proposed algorithm is based on computing the inverse fast Fourier transform (IFFT) of a desired target focusing profile, which is then binarized in order to obtain the governing binary function of the bifocal ZP. Bifocal focusing profiles can be useful in several fields, such as acoustic trapping applications [29,30] or therapeutic applications involving tumor ablation [31]. Focusing profiles with two foci can be useful to treat either isolated areas simultaneously, or even larger areas when the two foci are close enough. On the other hand, multi-foci profiles can enhance the complexity patterns in acoustic trapping.

The design technique is analyzed using numerical simulations for the ultrasound domain. The range of ultrasound frequencies is similar to that of conventional ultrasound Fresnel ZPs, and can vary typically between 100 kHz and 20 MHz. Although not shown in the paper, the results can be extended to other domains such as optics and microwaves, because the relation between the binary sequence and the ZP focusing profile is the same with independence of the domain.

## 2. Relation between ZP Transmittance Function and Focusing Profile

**Figure 1:**
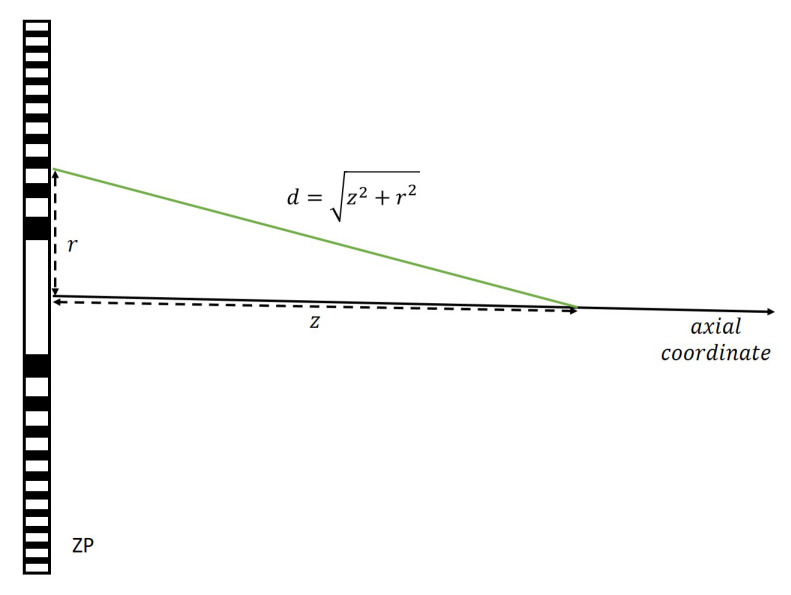
Schematic of the zone plate focusing procedure.

It is well known that, in a general zone plate, the acoustic intensity along the *z*-axis can be calculated by numerically computing the Rayleigh-Sommerfeld diffraction integral [32],

(1) $$I\left(z\right)=\frac{4\pi^{2}}{\lambda^{2}}{\left|\int_{0}^{a}p_{i}\left(r\right)t\left(r\right)\frac{e^{-jkd}}{d}\cos\left(n,d\right)rdr\right|}^{2},$$

where *a* is the maximum radius of the lens, *r* is the radial coordinate of the lens, k=2π/λ is the wavenumber, $p_{i}\left(r\right)$ is the incident pressure distribution, t(r) is the ZP transmittance function, $d=\sqrt{z^{2}+r^{2}}$, *z* is the axial coordinate, and cos(n,d)=z/d, with *n* being the normal direction to the lens surface. Figure 1 shows the schematic of the zone plate focusing procedure.

In Equation (1), the distance *d* can be expressed as,

(2) $$d=\sqrt{z^{2}+r^{2}}=z\sqrt{1+{\left(\frac{r}{z}\right)}^{2}}.$$

Using the Taylor expansion $\sqrt{1+x}\approx 1+x/2-x^{2}/8$, *d* can be approximated as,

(3) $$d\approx z+\frac{r^{2}}{2z}-\frac{r^{4}}{8z^{3}}.$$

The Fresnel approximation assumes that the third term of the Taylor expansion of *d* does not affect the phase of the exponential term, which means that its contribution to the exponent has to be much lower than 2π, that is,

(4) $$k\frac{r^{4}}{8z^{3}}<<2\pi .$$

The worst case is obtained when *r* reaches its maximum value at r=a, which means that the Fresnel approximation stands for axial coordinates greater than

(5) $$z^{3}>>\frac{a^{4}}{8\lambda }.$$

If the above condition is met, $d\approx z+\frac{r^{2}}{2z}$, and Equation (1) can be approximated as

(6) $$I\left(z\right)\approx \frac{4\pi^{2}}{\lambda^{2}}{\left|\int_{0}^{a}p_{i}\left(r\right)t\left(r\right)\frac{e^{-jkz}e^{-jk\frac{r^{2}}{2z}}}{z+\frac{r^{2}}{2z}}\cos\left(n,d\right)rdr\right|}^{2}.$$

Further approximations can be done, as the paraxial approximation is valid (z>>r) and thus, cos(n,d)≈1 and the denominator inside the integral becomes $z+\frac{r^{2}}{2z}\approx z$, which results in

(7) $$I\left(z\right)\approx \frac{4\pi^{2}}{\lambda^{2}z^{2}}{\left|\int_{0}^{a}p_{i}\left(r\right)t\left(r\right)e^{-jk\frac{r^{2}}{2z}}rdr\right|}^{2}.$$

We now make coordinate transformations in both axial and radial directions to work with the normalized domains, as previously stated in [19]. Thus, we define $u=\frac{a^{2}}{2\lambda z}$ as the normalized axial coordinate, whereas $\xi ={\left(\frac{r}{a}\right)}^{2}$ is defined as the normalized radial coordinate. Keeping in mind that $dr=\frac{a}{2\sqrt{\xi }}d\xi$, Equation (7) becomes

(8) $$I\left(u\right)\approx 4\pi^{2}u^{2}{\left|\int_{0}^{1}p_{i}\left(\xi \right)t\left(\xi \right)e^{-j2\pi \xi u}d\xi \right|}^{2}.$$

Thus, if the Fresnel approximation condition is fulfilled, the focusing profile of the lens along the normalized axial coordinate *u* can be obtained as a function of the Fourier transform of the pressure distribution at the ZP aperture. Moreover, if plain wave excitation is considered ($p_{i}\left(\xi \right)=1$), the focusing profile can be directly computed from the Fourier transform of the transmission function t(ξ),

(9) $$I\left(u\right)\approx 4\pi^{2}u^{2}{\left|\int_{0}^{1}t\left(\xi \right)e^{-j2\pi \xi u}d\xi \right|}^{2}.$$

All the assumptions considered to obtain Equation (9) are always valid for short wavelength domains such as optics, whereas, in long wavelength domains, such as acoustics or microwaves, the calculation of the ZP focusing profile through the Fourier transform approach can introduce a significant error, depending on the parameters of the design. Therefore, the use of the Fourier transform approximation is not always valid in long wavelength domains, and has to be used with caution if necessary.

## 3. Application of the Fast Fourier Transform to the Lens Binary Sequence

Once established that the lens transmittance function is related to the lens focusing profile, in normalized coordinates, through the Fourier transform, it becomes intriguing whether this mathematical tool can be used to improve the design process of ZP lenses based on binary sequences. The ZP transmittance function is governed by the lens binary sequence. From now on in this paper, phase-reversal ZPs (PRZPs) are always considered, although the results are equally applicable to Soret ZPs (SZPs). In a PRZP, the levels of the transmittance function are either 1, for transparent Fresnel regions, or −1, for phase-reversal Fresnel regions, values, which correspond to the binary sequence symbols “1” and “0”, respectively.

As the lens transmittance function is so closely related to the ZP governing binary sequence, it is interesting to analyze the result of directly applying the Fourier transform on the ZP binary sequence, b(n). Although the values more commonly used for representing a binary sequence are “1” and “0” as stated above, a different notation is going to be used through this paper in order to ease the presentation of the different results obtained when Fourier transforms are applied on ZP binary sequences, by removing the DC components. It is worth noting that b(n) is still a binary sequence as it only presents two possible different values, either a “1”, for transparent Fresnel regions, or a “−1” for phase-reversal Fresnel regions. In other words, the binary sequence is going to be represented with the same levels (“1” and “−1”) as the ZP transmittance function. However, the more conventional notation (“1” and “0”) will still be used for writing the different binary sequences with digits. For example, a sequence of 10 digits for a conventional Fresnel ZP will be described as b(n)={1010101010}, although it will be implemented as [1,−1,1,−1,1,−1,1,−1,1,−1], when depicted in a graphic or involved in a mathematical calculation such as the fast Fourier transform algorithm.

If the fast Fourier transform (FFT) is performed on the ZP binary sequence with *N* points, a new discrete sequence, F(n), is obtained in the transformed domain. A capital letter is used to denote this sequence in order to highlight the fact that this sequence belongs to the transformed domain. As it will be shown below, this discrete sequence F(n) resembles the ZP focusing profile, indicating the position of the different foci, and for this reason, this new sequence has been named as the focusing profile sequence. As this F(n) sequence conveys information about the ZP focusing profile, it is going to be normally depicted against the normalized axial coordinate *u*, for comparison purposes with the focusing profile of the actual lens. Therefore, a discrete $u_{n}$ sequence is defined to be used in conjunction with the F(n) sequence. This $u_{n}$ sequence is given by,

(10) $$u_{n}=0,1,2,\ldots N-1,$$

with N being the length of both the binary sequence, b(n), and the focusing profile sequence, F(n). The first value of the $u_{n}$ sequence is “0”, analogously to more conventional signal processing applications, where the first value of the frequency axis after an FFT is also “0”. As stated above, the sequence F(n) is going to be represented in this work against the $u_{n}$ sequence, and therefore the notation $F(u_{n})$ is going to be used from now on.

**Figure 2:**
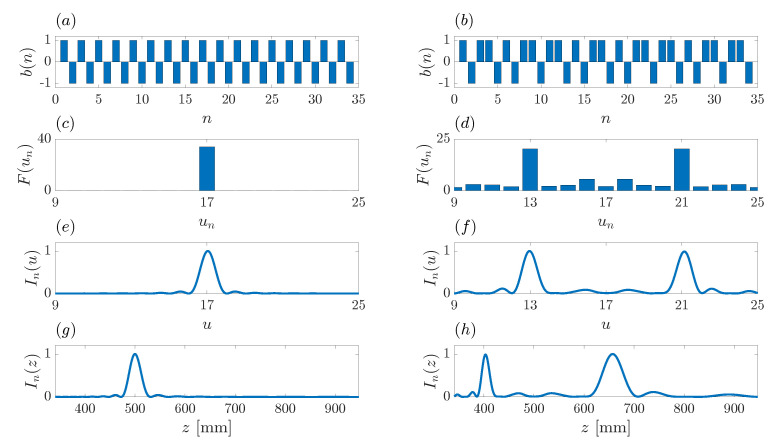
Fourier transforms on binary sequences for a Fresnel ZP (**a**,**c**,**e**,**g**) and a Fibonacci ZP (**b**,**d**,**f**,**h**). Binary sequences: (**a**,**b**). Focusing profile sequences: (**c**,**d**). Normalized focusing profile as a function of *u*: (**e**,**f**). Normalized focusing profile as a function of *z*: (**g**,**h**). The F(n) sequence is computed as the FFT of the b(n) sequence. Focusing profiles are computed using the Rayleigh-Sommerfeld diffraction integral.

As an example, Figure 2a,b show the binary sequences corresponding to a conventional Fresnel ZP (Figure 2a) and a Fibonacci ZP (Figure 2b). Both binary sequences are of length N=34. The Fresnel ZP binary sequence is an alternating sequence of “1” and “0” values, given by b(n)={1010101010101010101010101010101010}, whereas b(n)={1011010110110101101011011010110110} is the Fibonacci binary sequence of order j=8 depicted in Figure 2b. As can be observed from Figure 2a,b, the values used to implement the different binary sequences are “1” and “−1” as stated above, instead of the more conventional notation of “1” and “0”, which is still used to provide the b(n) sequences in writing.

Figure 2c,d show the $F(u_{n})$ focusing profile sequences obtained after applying the FFT to each binary sequence. Figure 2c presents a discrete sequence with a significant value around its middle position ($u_{n}=17$), whereas Figure 2d depicts a discrete sequence with two main values ($u_{n}=13$ and $u_{n}=21$), evenly distributed around the central location ($u_{n}=17$). The F(n) behavior is coherent with the number of foci that are obtained in this type of ZPs. A conventional Fresnel ZP presents a single focus, whereas a Fibonacci ZP presents two foci of similar level.

In order to deepen in this analysis, we have computed the ZP focusing profiles for both binary sequences (Fresnel ZP and Fibonacci ZP). These focusing profiles have been calculated by computing the Rayleigh-Sommerfeld diffraction integral, and have been depicted against both the normalized axial coordinate, *u*, and the denormalized axial coordinate, *z*. A frequency of f=10 MHz has been considered in the simulations, while the central focus position has been set to $z_{0}=500$ mm. Figure 2g,h depict the ZP normalized focusing profiles $I_{n}\left(z\right)$ vs. the *z* coordinate for the Fresnel ZP and the Fibonacci ZP, respectively. As can be observed from these figures, the Fresnel ZP presents a single focus at z=500 mm, whereas the Fibonacci ZP presents two foci of similar magnitude at z=403 mm and z=657 mm, respectively. These results match the design parameters of both ZPs, as expected.

The interesting results arise when the focusing profiles are represented against the normalized axial coordinate, *u*. Figure 2e,f depict the ZP normalized focusing profiles, $I_{n}\left(u\right)$, for the Fresnel ZP and the Fibonacci ZP, respectively. As can be observed when comparing Figure 2c,e, the Fresnel ZP focusing profile, when depicted as a function of *u*, has a very similar behavior as that of the $F(u_{n})$ sequence, that is generated by direct application of the FFT to the Fresnel ZP binary sequence b(n). Not only is there a similar behavior, but the main focus appears at the exact same location in both cases. The main focus at the Fresnel ZP focusing profile is located at u=17, whereas $u_{n}=17$ is the location of the highest value in the $F(u_{n})$ sequence. The same conclusion can be obtained in the Fibonacci ZP case, comparing Figure 2d,f. In this case, the ZP focusing profile presents two foci of similar magnitude located at u=13 and u=21, which corresponds to the locations $u_{n}=13$ and $u_{n}=17$ at which the $F(u_{n})$ sequence presents its highest values.

Therefore, we can conclude from Figure 2 that by applying the FFT to the ZP binary sequence, b(n), we obtain a discrete focusing profile sequence, $F(u_{n})$, which accurately resembles the actual ZP focusing profile, providing information on the number and location of the different foci.

**Figure 3:**
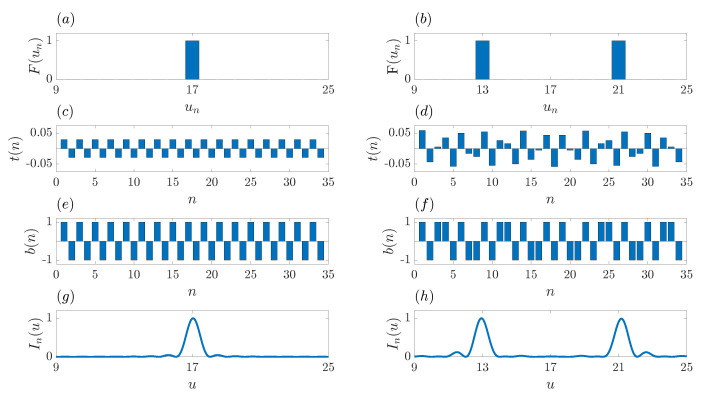
Generation of the ZP binary sequence for a monofocal focusing profile (**a**,**c**,**e**,**g**) and a bifocal focusing profile (**b**,**d**,**f**,**h**). Target focusing profiles: (**a**,**b**). Transmittance sequences: (**c**,**d**). Binary sequences: (**e**,**f**). Normalized focusing profiles, computed with the Rayleigh-Sommerfeld diffraction integral, as a function of *u*: (**g**,**h**).

Applying reverse engineering, we can investigate now what kind of discrete sequence is going to be generated if a target focusing profile is artificially generated, and then the inverse fast Fourier transform (IFFT) is applied. Going in the backwards direction, it is possible to imagine that the procedure could end with the generation of a binary sequence suitable for the design of a ZP that would provide the target focusing profile. Figure 3a,b depict the $F(u_{n})$ sequences that correspond to a monofocal and bifocal focusing profile, respectively. These $F(u_{n})$ sequences are designed with the same number of elements as the number of Fresnel regions that is required for the target ZP. It will be shown later on, that the length of the $F(u_{n})$ sequence is closely related with the resolution of the focusing profile of the target ZP, as expected, because FZPs with a higher number of Fresnel regions provide higher resolutions. Thus, the $F(u_{n})$ sequence is set to “0”, except those positions where the foci are located, which are set to “1”.

Working with even sequences is very helpful when operating with FFTs and IFFTs. We refer to an even sequence as a sequence that provides symmetry around its central location and has an even number of elements. When applying the IFFT algorithm to an even sequence, the output sequence is always going to be real with no imaginary components, which in turns enables this sequence as a potential tool for designing a ZP with a desired focusing profile. Thus, it is fundamental to distribute the foci of the desired target profile in an symmetrical pattern. Both Figure 3a,b depict even sequences that fulfill the symmetry requirement, and therefore, after the IFFT is applied to both sequences, the resulting output sequences only have real components with no imaginary part. Figure 3c,d show the sequences obtained after applying the IFFT to the target profiles. Although the monofocal case (Figure 3c) depicts a discrete sequence with only two values, in general a multivalue sequence is going to be generated as shown in the bifocal case (Figure 3d). Thus, this multivalue sequence is not going to be a binary sequence, and therefore, we are going to refer to it as the transmittance sequence, t(n), that would be ideally implemented to design a ZP with the desired target focusing profile. As this t(n) sequence cannot be directly implemented in the design of ZPs, it is going to be submitted to a binarization process, with the result being a binary sequence, b(n), with only two allowed values (“1” and “−1”), that can then be successfully used to design a ZP. The binary sequence b(n) is obtained from the transmittance sequence t(n) applying the following threshold condition,

(11) $$b\left(n\right)=\left\{\begin{array}{cc} 1; & t(n)\geq 0\\ -1; & t(n)<0. \end{array}\right.$$

Figure 3e,f depict the binary sequences generated for the monofocal and bifocal cases, respectively. As can be observed from Figure 3e, the generated binary sequence perfectly matches the binary sequence of a conventional Fresnel ZP (Figure 2a). Therefore, the focusing profile of the ZP designed with this b(n) sequence is in fact a monofocal ZP with the focus at the desired location as shown in Figure 3g. Although the generated b(n) sequence for the bifocal case (Figure 3f) does not exactly match the Fibonacci sequence (Figure 2b), a ZP design with this binary sequence also provides a bifocal focusing profile (Figure 3h) with the foci locations at the expected positions. Therefore, we can conclude that the application of the IFFT algorithm on desired target profiles, allows the generation of binary sequences that can be successfully employed for the design of ZPs with tailored focusing profiles. This fact is going to be the seed for the design algorithm presented in the next section.

## 4. Fourier-Based Design Algorithm

Because of the even symmetry condition introduced in the previous section and simplicity constrains, the design algorithm is going to be implemented for the generation of tailored bifocal focusing profiles, with two foci of the same intensity levels at predetermined locations $z_{1}$ and $z_{2}$. Moreover, focal resolution is also a critical parameter and is going to be controlled through the length of the binary sequence, *N*. Finally, another fundamental parameter is either the design frequency (*f*), or alternatively the design wavelength (λ), of the ZP lens. Thus, the input parameters of the design algorithm are then $z_{1}$, $z_{2}$, *f* and *N*.

*Step 1: Initial Calculations*

The first step consists of performing some simple calculations to obtain $u_{1}$ and $u_{2}$, i.e., the axial locations of both foci in the normalized axial domain *u*. With these parameters and the length of the binary sequence (*N*) the target focusing profile can be easily implemented in the next step.

The parameter $u_{0}$ corresponds to the intermediate position between $u_{1}$ and $u_{2}$. As previously stated, in order to obtain a binary sequence with only real components, the focusing profile sequence is required to be even, and thus $u_{0}$ must be the central location,

(12) $$u_{0}=\frac{N}{2},$$

Then, $z_{0}$, which stands for the corresponding axial location in the denormalized axial domain, can be easily obtained from the input parameters $z_{1}$, $z_{2}$ and *f* by means of

(13) $$z_{0}=\frac{a^{2}}{2\lambda u_{0}}=\frac{a^{2}}{2\lambda \frac{u_{1}+u_{2}}{2}}=\frac{a^{2}}{\lambda \left(\frac{a^{2}}{2\lambda z_{1}}+\frac{a^{2}}{2\lambda u_{2}}\right)}=\frac{2z_{1}z_{2}}{z_{1}+z_{2}},$$

with λ=c/f and being *c* the speed of sound.

It should be pointed out that in a monofocal conventional Fresnel ZP design with the same design parameters (*N* and *f*), $u_{0}$ and $z_{0}$ correspond to the location of the main focus at the lens focusing profile in the normalized and denormalized axial coordinate, respectively. Once $u_{0}$ and $z_{0}$ are obtained, the size of the lens, *a*, can then be calculated as

(14) $$a=\sqrt{2\lambda u_{0}z_{0}}.$$

Finally, the locations of the foci in the normalized axial domain can be easily obtained with

(15) $$\begin{array}{c} u_{1}=\frac{a^{2}}{2\lambda z_{1}}\\ u_{2}=\frac{a^{2}}{2\lambda z_{2}}. \end{array}$$

It must be noted that $u_{1}$ and $u_{2}$ must be rounded to their closest integers if necessary, in order for the foci to properly fit in the focusing profile sequence $F(u_{n})$.

*Step 2: Generation of the focusing profile sequence $F(u_{n})$*

With the information provided by $u_{1}$ and $u_{2}$, the focusing profile sequence F(n) can now be designed as a binary sequence of length *N*, with all its elements equal to 0, except those corresponding to the axial normalized coordinates $u_{1}$ and $u_{2}$, which are set to a value of 1 and indicate the desired locations of the foci during the ZP design.

*Step 3: Generation of the target transmittance sequence t(n)*

Applying the IFFT algorithm to the focusing profile sequence, a target transmittance sequence t(n) is obtained, which only has real components, as it is built from an even sequence in the transformed domain.

(16) t(n)=IFFT{F(n)},

where n=1,2,…,N and IFFT stands for the inverse fast Fourier transform.

*Step 4: Generation of the design binary sequence b(n)*

This transmittance sequence t(n) is now binarized (b(n)) using the threshold condition shown in Equation (11). This is the final result of the design algorithm: the generation of the binary sequence, b(n), required to implement the ZP lens that will provide the target bifocal focusing profile with foci locations at $z_{1}$ and $z_{2}$.

Once the binary sequence has been obtained, it is applied to the Fresnel regions of a conventional monofocal Fresnel ZP to generate the new bifocal ZP. The binary sequence simply indicates, for each Fresnel region, whether that region is either transparent or phase-reversal. For high frequency scenarios and under plane wave incidence, Fresnel regions are defined through the Fresnel radii, which can be calculated as

(17) $$r_{n}=\sqrt{n\lambda z_{0}}.$$

## 5. Simulation Results and Discussion

The design algorithm presented in the previous section can be used as a powerful and easy-to-use tool to tailor ZP focusing profiles to the specific requirements of any given application. The following example shows the design of a bifocal ZP with foci locations at $z_{1}=400$ mm and $z_{2}=600$ mm. The length of the binary sequence is set to N=40 and the design frequency is f=10 MHz. Underwater ultrasound propagation has been assumed with c=1500 m/s the speed of sound in water.

**Figure 4:**
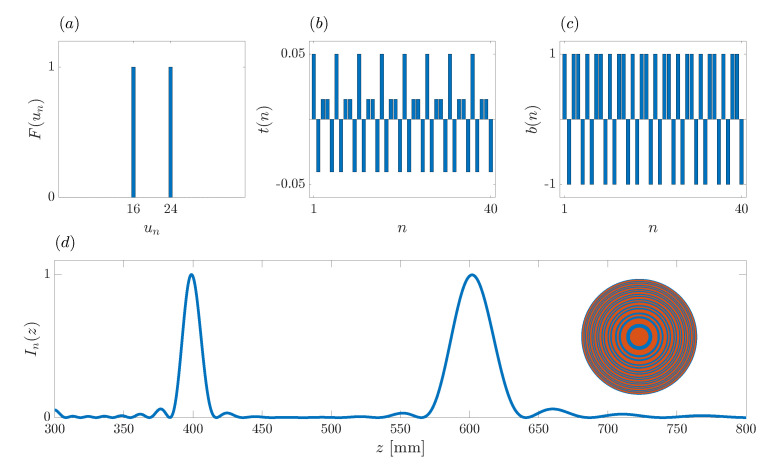
Example of the design of a bifocal ZP with foci locations at z1=400 mm and z2=600 mm. (**a**) Focusing profile sequence. (**b**) Transmittance sequence. (**c**) ZP binary sequence. (**d**) ZP bifocal normalized focusing profile. Inset: Bifocal ZP layout with transparent areas (red) and phase-reversal areas (blue).

- Step 1: The first step consists of performing the necessary calculations to obtain the $u_{1}$ and $u_{2}$ parameters, using Equations (12)–(15).
  (18) $$\begin{array}{ccc} \lambda & = & 0.15\;\mathrm{mm}\\ u_{0} & = & 20\\ z_{0} & = & 480\;\mathrm{mm}\\ a & = & 53.7\;\mathrm{mm}\\ u_{1} & = & 24\\ u_{2} & = & 16 \end{array}$$
  As $u_{1}$ and $u_{2}$ are integer numbers, the first step is already finished;
- Step 2: The focusing profile sequence, $F(u_{n})$, is generated using parameters *N*, $u_{1}$ and $u_{2}$. Figure 4a depicts the target focusing profile, where the two foci can be sharply distinguished;
- Step 3: Applying the IFFT to the focusing profile sequence, the transmittance sequence, t(n), is generated as shown in Figure 4b;
- Step 4: Figure 4c depicts b(n), the result of binarizing the transmittance sequence. With this final step, the design process is completed. In this particular case, the generated binary sequence is b(n)={1011010110101101011010110101101011010110}.

In order to verify the performance of the proposed algorithm, Figure 4d depicts the actual focusing profile of the ZP lens designed according to the synthesized binary sequence b(n). Additionally, the inset shows the corresponding ZP layout, where red and blue colors have been used to depict the ZP transparent and phase-reversal regions, respectively. Thus, red areas let the acoustic signal go through without further effect, whereas blue regions introduce a 180${}^{\circ }$ phase shift on the acoustic signal. The combination of the acoustic signals from the different regions produces the depicted focusing profile. Although in this example N=40, the number of different rings at the ZP layout is lower, because some Fresnel regions of the same color are adjacent, and thus, combined in thicker rings. As can be observed from Figure 4d, the foci are around the expected locations, $z_{1}=400$ mm and $z_{2}=600$ mm, and the focal intensity of both foci is evenly distributed. Therefore, the design requirements have been successfully achieved.

**Figure 5:**
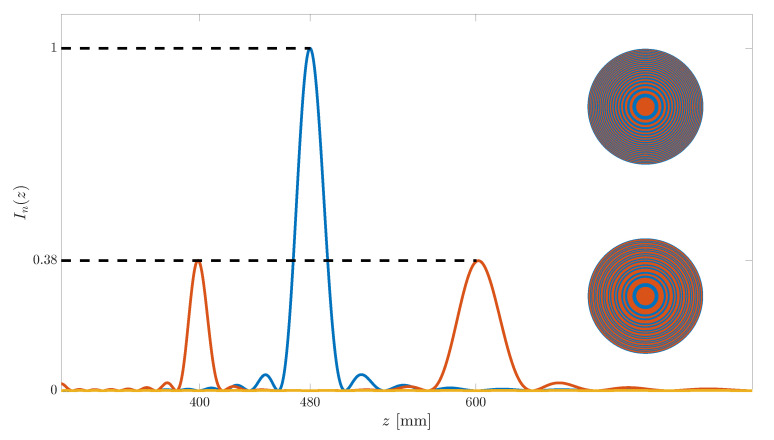
Normalized focusing profiles vs. axial coordinate for the conventional Fresnel ZP (blue), bifocal ZP (red) and no lens (yellow) cases. Insets depict the ZP layouts for the Fresnel ZP (top) and bifocal ZP (bottom).

Figure 5 depicts several focusing profiles for comparison purposes. The red line corresponds to the bifocal ZP example depicted in Figure 4, whereas the blue line depicts the focusing profile of the monofocal conventional Fresnel ZP that corresponds to the same design parameters (λ, $z_{0}$, *N*) used in that example. Finally, as a reference, the yellow line depicts the acoustic intensity along the longitudinal axis when no lens is employed. As expected, there are no foci along the focusing profile when operating without a lens. The top inset shows the layout of the conventional Fresnel ZP that generates the monofocal focusing profile, whereas the bottom inset depicts the layout of the bifocal ZP. All focusing profiles are normalized to the maximum value, which corresponds to the main focus of the monofocal profile. The acoustic intensity at both foci in the bifocal focusing profile is around 38% of the maximum value for the monofocal case, as the acoustic energy is split between two foci in the bifocal profile.

**Figure 6:**
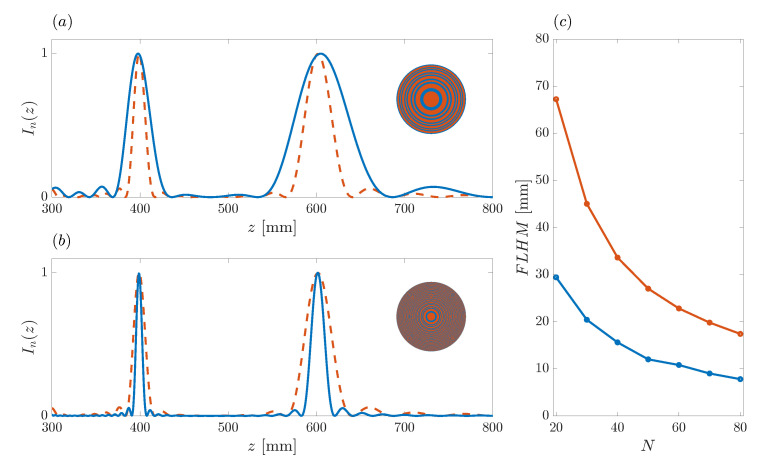
Bifocal ZP focusing profiles vs. axial coordinate. Red dashed line: N=40. Blue solid line: (**a**) N=20 and (**b**) N=80. Insets depict the ZP layouts for N=20 (top) and N=80 (bottom). (**c**) FLHM at the first (blue) and second (red) focus against *N*.

Figure 6 shows the effect of the variation of the binary sequence length *N* on the ZP focusing profile. The blue solid lines of Figure 6a,b correspond to normalized focusing profiles generated from binary sequences with N=20 and N=80, respectively, whereas the red dashed line that appears in both subplots corresponds to the design example of the previous section (N=40). For comparison purposes, only the *N* parameter has been modified among the different cases. As can be observed from the figure, the longitudinal resolution of the ZP focusing profile is inversely proportional to the length of the binary sequence, as expected. The longer the binary sequence, the higher the number of Fresnel regions that contribute to the main focus, and thus, the better the resolution. The lowest longitudinal resolution is obtained in the N=20 case (blue line in Figure 6a), whereas the highest resolution corresponds to the narrowest foci depicted with the blue curve in Figure 6b (N=80). However, although the resolution is modified through the *N* parameter, the location of both foci remains unchanged at the same locations $z_{1}=400$ mm and $z_{2}=600$ mm. The ZP layouts corresponding to the focusing profiles depicted with the blue solid lines are shown at the corresponding insets. Figure 6c depicts the FLHM parameters for both foci as a function of the number of Fresnel regions used to implement the bifocal ZP. FLHM stands for ‘Full Length Half Maximum’, corresponds to the length of the focus in the axial direction, and is calculated from the difference between the *z* locations, at opposite sides of the focus central point, at which the normalized intensity is half of its maximum value. The blue and red lines depict the FLHM parameter for the first (${\mathrm{FLHM}}_{1}$) and second (${\mathrm{FLHM}}_{2}$) focus, respectively. As can be observed from the figure, the resolution augments (lower FLHM) for both foci when N is increased. It can also be stated that the second focus always has a lower resolution than the first one for the same *N* parameter, although the resolution of the second focus can be higher than that of the first focus for different *N* values. For example ${\mathrm{FLHM}}_{1}=29.43$ mm for N=20, whereas ${\mathrm{FLHM}}_{2}=17.42$ mm for N=80, which can also be graphically deduced by comparing the blue lines at the first focus in Figure 6a (N=20) with the second focus in Figure 6b (N=80). Table 1 shows the FLHM parameters for the foci from Figure 6a,b. As can be observed from the table, when the number of Fresnel regions is doubled, the FLHM parameter is approximately halved at both foci. The last column of Table 1 shows the ratio between the FLHM values of both foci. This ratio remains quite steady with *N*.

**Figure 7:**
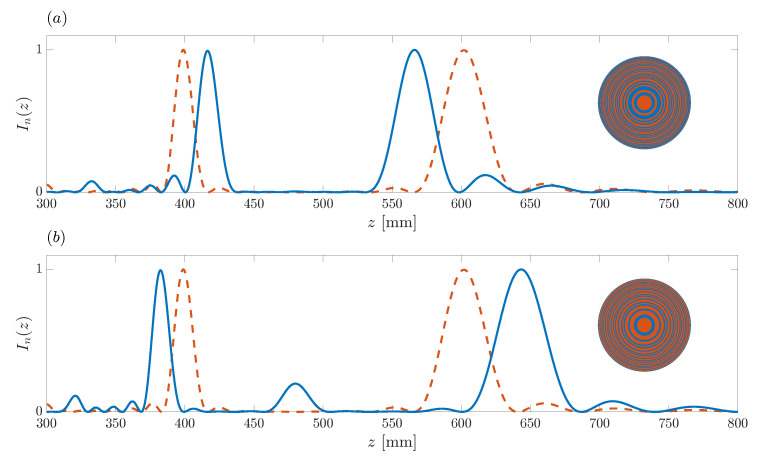
Bifocal ZP focusing profiles vs. axial coordinate. Blue solid line (**a**) $u_{1}=24$ and $u_{2}=18$ (inwards shift case), (**b**) $u_{1}=26$ and $u_{2}=16$ (outwards shift case). Dashed red line corresponds to the initial design example with $u_{1}=25$ and $u_{2}=17$. Insets depict the ZP layouts for the focusing profiles depicted in blue.

Figure 7 shows the effect of shifting the foci locations by one position in the target focusing profile sequence. The red dashed line is the reference case and corresponds to the ZP designed in the previous example, with design parameters N=40, f=10 MHz, $z_{1}=400$ mm, and $z_{2}=600$ mm ($u_{1}=25$ and $u_{2}=17$). However, before applying the inverse Fourier transform to $F(u_{n})$ in this analysis, the foci locations are shifted one position inwards ($u_{1}=24$ and $u_{2}=18$) or one position outwards ($u_{1}=26$ and $u_{2}=16$) in order to study the effect of these variations on the ZP focusing profile. Figure 7a,b depict the ZP focusing profiles, using blue solid lines, for the inward and outward cases, respectively, whereas the dashed red line depicts the reference case where the foci locations have not been modified. The insets show the ZP layouts for the modified focusing profiles in each case. As expected, the actual locations of the two foci are also shifted in the same direction, either inwards or outwards, as in the F(n) sequence. The difference between the blue and red curves in each subplot roughly indicates the minimum axial displacement that can be introduced to each focus. If the length of the binary sequence *N* is increased, the lateral resolution becomes higher, as has been indicated in Figure 6, but the size of the minimum axial displacement will also be reduced, providing higher accuracy.

**Figure 8:**
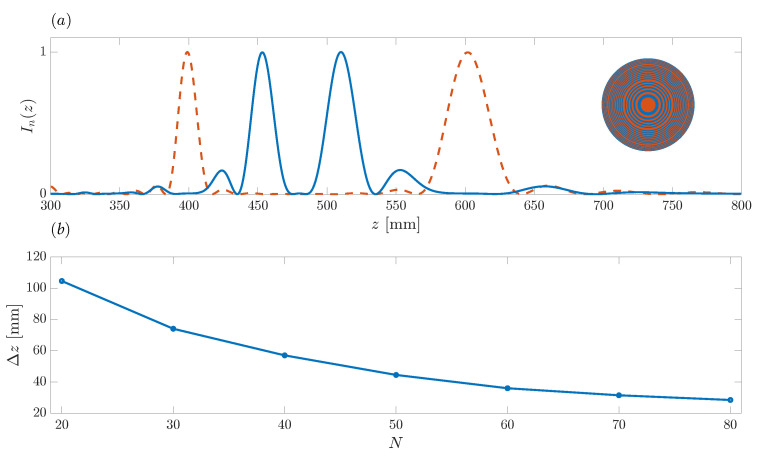
(**a**) Bifocal ZP focusing profiles vs. axial coordinate. Blue solid line: resolution limit case with $u_{1}=22$ and $u_{2}=20$. Dashed red line: initial design example with $u_{1}=25$ and $u_{2}=17$. Inset depict the ZP layout for the resolution limit case. (**b**) Resolution limit against *N*.

Figure 8a shows the effect of inwards shifting the foci locations at its maximum in order to obtain the resolution limit Δz. As in Figure 7, the red dashed line is the reference case and corresponds to the ZP designed in the previous example, with design parameters N=40, f=10 MHz, $z_{1}=400$ mm, and $z_{2}=600$ mm ($u_{1}=25$ and $u_{2}=17$). The blue line corresponds to the ZP designed by shifting the foci locations inwards as much as possible ($u_{1}=22$ and $u_{2}=20$), before applying the inverse Fourier transform to $F(u_{n})$. The foci locations are in this case $z_{1}=453.15$ mm and $z_{2}=510.21$ mm, resulting in a resolution limit of Δz=57.06 mm. Figure 8b shows the resolution limit against *N*. As expected, when *N* increases, the bifocal ZP presents a higher resolution limit, i.e., a lower Δz parameter. The trend is very similar to that shown in Figure 6c, and the Δz parameter performs similarly to the FLHM parameter. Thus, when the *N* doubles, the Δz parameter is approximately halved, as was the FLHM parameter, improving then the resolution limit. For example, N=80 corresponds to a resolution limit of Δz=28.58 mm.

**Figure 9:**
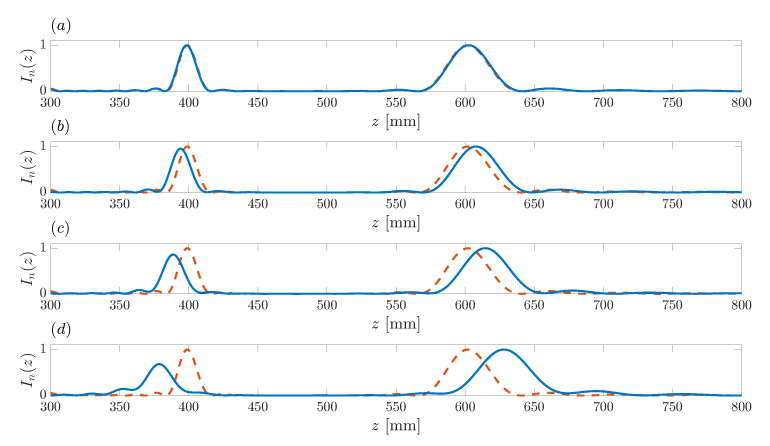
Bifocal ZP focusing profiles vs. axial coordinate for different design frequencies. Blue solid line: (**a**) f=5 MHz, (**b**) f=1 MHz, (**c**) f=500 kHz, (**d**) f=250 kHz. Dashed red line corresponds to the initial design example with f=10 MHz.

Figure 9 depicts ZP focusing profiles for different design frequencies. As stated in Section 3, the approximation based on the Fourier transform that can be used to compute the ZP focusing profile is not always accurate in long wavelength domains. Thus, it seems interesting to investigate how the design wavelength, or alternatively the design frequency, affects the proposed algorithm. The blue lines depicted in Figure 9a–d correspond to ZP focusing profiles for design frequencies f=5 MHz, f=1 MHz, f=500 kHz, and f=250 kHz, respectively. The red dashed line that is depicted in each subplot corresponds to the reference case at f=10 MHz. As can be observed from Figure 9a, the algorithm performs very well up to frequencies as low as 5 MHz, as there is not a significant difference between the f=10 MHz and the f=5 MHz cases. Figure 9b shows a significant difference between the f=10 MHz and the f=1 MHz cases, with a certain distortion that produces a slight displacement in the foci positions, as well as a slight reduction on the level of the first focus. This distortion is further aggravated in the f=500 kHz and f=250 kHz cases, as can be observed from Figure 9c,d. It should be noted that this issue is only significant when operating in long wavelength domains, such as acoustics or microwaves. In optics, the design algorithm presented in this work would always perform successfully.

**Figure 10:**
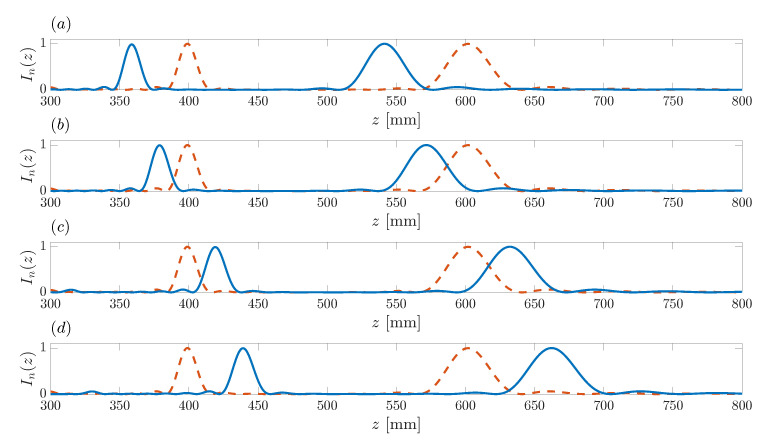
Bifocal ZP focusing profiles vs. axial coordinate for different operating frequencies. Blue solid line: (**a**) f=9 MHz, (**b**) f=9.5 MHz, (**c**) f=10.5 MHz, (**d**) f=11 MHz. The dashed red line corresponds to an operating frequency equal to the design frequency (f=10 MHz).

Once the design frequency has been fixed at a proper value, in our design example f=10 MHz, the operating frequency at which the ZP is being employed can be shifted from its design counterpart. The shift in the operating frequency results in controlled foci displacement as it has already been shown in monofocal conventional Fresnel ZPs [33] and bifocal Fibonacci ZPs [21]. Figure 10 depicts ZP focusing profiles for different operating frequencies. In all cases, the design frequency is f=10 MHz. The red dashed line depicts the ZP focusing profile achieved when the operating frequency matches the design frequency. The blue solid lines in Figure 10a–d depict the ZP focusing profiles achieved for operating frequencies f=9 MHz, f=9.5 MHz, f=10.5 MHz, and f=11 MHz, respectively. As can be observed from the figure, an increase in the operating frequency results in a focal displacement where both foci move further away from the ZP plane. On the contrary, both foci move towards the lens when the operating frequency is diminished. Therefore, the shift in the operating frequency can be used to finely adjust the location of the foci, or as a dynamic mechanism in order to have real-time focal shifting that can be very helpful in certain applications.

**Figure 11:**
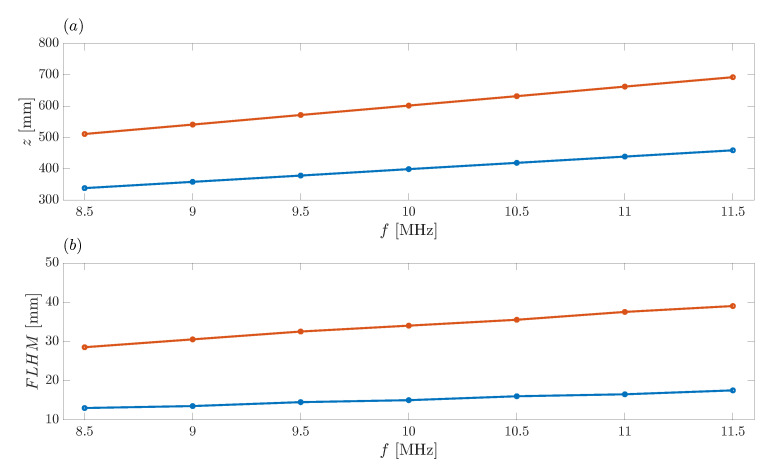
(**a**) Foci location against operating frequency. (**b**) Foci FLHM against operating frequency. Blue and red lines refer to the first and second focus at the bifocal focusing profile, respectively.

Figure 11a shows the variation of the foci locations as a function of the operating frequency, whereas Figure 11b shows the variation of the FLHM parameter for both foci as a function of the operating frequency. Blue and red lines correspond to the first and second focus of the bifocal focusing profile, respectively. As can be observed from the figure, all four curves present a linear dependence, and thus, the operating frequency is an interesting and easy-to-use parameter to dynamically shift the focal locations or the focal resolutions in real-time applications, if necessary.

**Figure 12:**
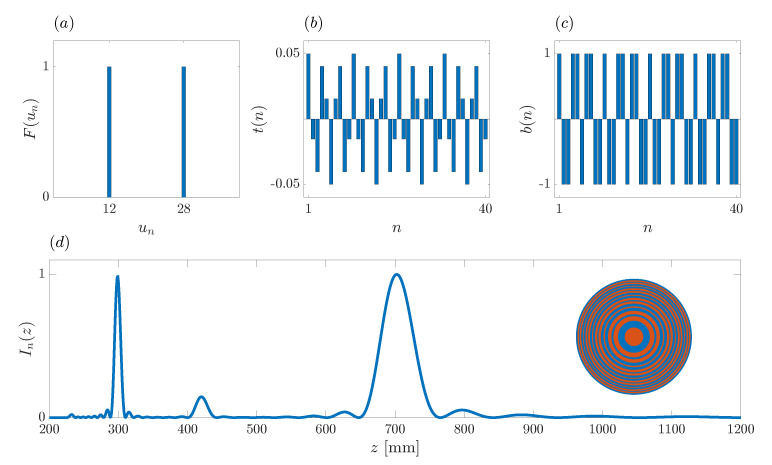
Example of the design of a bifocal ZP with foci locations at z1=300 mm and z2=700 mm. (**a**) Focusing profile sequence. (**b**) Transmittance sequence. (**c**) ZP binary sequence. (**d**) ZP bifocal normalized focusing profile. Inset: Bifocal ZP layout.

Figure 12 depicts an additional design example with $z_{1}=300$ mm and $z_{2}=700$ mm. The rest of the design parameters remain unchanged from the previous example, with N=40 and f=10 MHz.

- Step 1: Calculations to obtain $u_{1}$ and $u_{2}$, using Equations (12)–(15).
  (19) $$\begin{array}{ccc} \lambda & = & 0.15\;\mathrm{mm}\\ u_{0} & = & 20\\ z_{0} & = & 420\;\mathrm{mm}\\ a & = & 50.2\;\mathrm{mm}\\ u_{1} & = & 28\\ u_{2} & = & 12 \end{array}$$
  As $u_{1}$ and $u_{2}$ are integer numbers, the first step is completed;
- Step 2: The focusing profile sequence, $F(u_{n})$, is generated using parameters *N*, $u_{1}$ and $u_{2}$. Figure 12a depicts the target focusing profile, where the two foci can be sharply distinguished;
- Step 3: Applying the IFFT to the focusing profile sequence, the transmittance sequence, t(n) is generated as shown in Figure 12b;
- Step 4: Figure 12c shows the result of binarizing the transmittance sequence, which ends the design process. In this design example, the generated binary sequence is b(n)={1001101100100110110010011011001001101100}.

Figure 12d depicts the actual focusing profile of the ZP lens designed according to the generated binary sequence. Additionally, the inset shows the corresponding ZP layout. As can be observed from Figure 12d, the foci are around the expected locations, $z_{1}=300$ mm and $z_{2}=700$ mm, and the focal intensity of both foci is evenly distributed, again successfully demonstrating the potential of this design algorithm.

Finally, it should be highlighted that the design method presented in this paper has been simulated for underwater ultrasonic applications, but it can also be used for designing ZPs in the optical or the microwave domain. Therefore, this design procedure can be of interest for many researchers working in different fields related to wave focusing applications.

## 6. Conclusions

In this work, we develop a design procedure based on the fast Fourier transform (FFT) algorithm for implementing zone plates (ZPs) with bifocal focusing profiles. This design algorithm is very easy to implement, and allows to tailor the design of bifocal ZPs to achieve focusing profiles with desired foci locations and resolutions. The algorithm has been thoroughly developed, and the effect of different design parameters on the ZP focusing profile has been analyzed in detail. The key advantage of this design algorithm is that it provides bifocal focusing profiles with an absolute control of the foci locations. Moreover, although we have shown the performance of this novel design algorithm for underwater ultrasonics, it can also be successfully implemented in different fields of physics, such as microwaves, x-rays, or optics, where ZPs are largely employed.

## Figures and Tables

**Table 1:** FLHM values for Figure 6a,b.

*N*	${\mathrm{FLHM}}_{1}$ [mm]	${\mathrm{FLHM}}_{2}$ [mm]	${\mathrm{FLHM}}_{2}/{\mathrm{FLHM}}_{1}$
20	29.43	67.27	2.29
40	15.62	33.63	2.15
80	7.81	17.42	2.23

## Data Availability

Data will be available upon reasonable request to the corresponding author.
